# High-purity Cu nanocrystal synthesis by a dynamic decomposition method

**DOI:** 10.1186/1556-276X-9-689

**Published:** 2014-12-20

**Authors:** Xian Jian, Yu Cao, Guozhang Chen, Chao Wang, Hui Tang, Liangjun Yin, Chunhong Luan, Yinglin Liang, Jing Jiang, Sixin Wu, Qing Zeng, Fei Wang, Chengui Zhang

**Affiliations:** Clean Energy Materials and Engineering Center, School of Energy Science and Engineering, University of Electronic Science and Technology of China, No. 2006, Xiyuan Avenue, West Hi-Tech Zone, Chengdu, 611731 China; State Key Laboratory of Electronic Thin Films and Integrated Devices, Center for Information in BioMedicine, University of Electronic Science and Technology of China, No. 4, Section 2, North Jianshe Road, Chengdu, 610054 China

**Keywords:** Cupric tartrate, Cu nanocrystals, Precursor decomposition, Chemical vapor decomposition

## Abstract

Cu nanocrystals are applied extensively in several fields, particularly in the microelectron, sensor, and catalysis. The catalytic behavior of Cu nanocrystals depends mainly on the structure and particle size. In this work, formation of high-purity Cu nanocrystals is studied using a common chemical vapor deposition precursor of cupric tartrate. This process is investigated through a combined experimental and computational approach. The decomposition kinetics is researched via differential scanning calorimetry and thermogravimetric analysis using Flynn-Wall-Ozawa, Kissinger, and Starink methods. The growth was found to be influenced by the factors of reaction temperature, protective gas, and time. And microstructural and thermal characterizations were performed by X-ray diffraction, scanning electron microscopy, transmission electron microscopy, and differential scanning calorimetry. Decomposition of cupric tartrate at different temperatures was simulated by density functional theory calculations under the generalized gradient approximation. High crystalline Cu nanocrystals without floccules were obtained from thermal decomposition of cupric tartrate at 271°C for 8 h under Ar. This general approach paves a way to controllable synthesis of Cu nanocrystals with high purity.

## Background

Metal nanocrystals with certain size and morphology have drawn great interests, not only due to their advanced chemical, electronic, optical, catalytic, and conductive properties but also on account of their wide applications in the fields of catalysts, sensors, optical devices, and so on [1–3]. With regard to the controllable synthesis of metal nanomaterial, most relevant studies have focused on Au and Ag nanocrystals because of their high conductivity and strong antioxidation properties [4–6]. However, potential large-scale use is limited greatly by the high cost of gold nanoparticles and the low resistivity toward ion migration of silver nanoparticles [7]. Therefore, it is necessary to explore an alternative that is inexpensive, highly purity, and environmental-friendly. Cu nanocrystal is a promising candidate in practical applications due to its excellent nonlinear optical properties, low bulk resistivity, good thermal stability, low-temperature coefficient of resistivity, etc. [8–11] The size and morphology of Cu nanocrystals can be controlled by lattice plane [12, 13]. There have been a number of methods proposed for preparing Cu nanocrystals, including physical vapor deposition [14], high-energy ball mill [15], γ radiation [16], chemical vapor deposition (CVD) [17], chemical precipitation [18], micro-emulsion [19], sol–gel [20], hydrothermal [21], electrolytic [22], and liquid-phase reduction methods [23]. However, these methods have many inherent problems. For instance, iron impurities cannot be removed completely using high-energy ball mill [24]. It is difficult to control the size of particles by CVD due to the high growth temperature [25]. Therefore, it is desired to explore new approaches to achieve Cu nanocrystals with good qualities. Herein, we propose a controllable, highly efficient, low-cost, and environmental-friendly approach where highly pure Cu nanocrystals can be synthesized from the decomposition of cupric tartrate precursor.

## Methods

### Experiment

Sodium potassium tartrate tetrahydrate (KNaC_4_H_4_O_6_•4H_2_O) and copper chloride were purchased from Tianjin Haijing Chemical Reagents Company, Tianjin, China, and Chengdu Jinshan Chemical Reagents Company Chengdu, China, respectively. High-purity gases including Ar and N_2_ were supplied by Chengdu Xuyuan Chemical Reagents Industry, Chengdu, China. All the reagents were analytical grade and used without further purification.

### The preparation of cupric tartrate and Cu nanocrystals

First, copper chloride solution (100 ml, 0.01 M) was dripped into sodium potassium tartrate tetrahydrate solution (100 ml, 0.01 M) under constant magnetic stirring. Slowly, light blue cupric tartrate precipitates appeared. After magnetic stirring of 0.5 h, the blue precipitates were filtered and subsequently washed for three times with distilled water and ethanol. And then, the cupric tartrate was purified via Soxhlet extraction for 1.5 h and dried at 110°C for 4 h. After drying, the cupric tartrate powders were calcined at temperatures ranging from 200°C to 400°C, under the protecting gas of Ar or N_2_.

### Characterizations

The thermal properties of cupric tartrate were investigated using a thermoanalyzers (TGA; STA 449C Jupiter, Selb, NETZSCH, Germany) under N_2_ atmosphere at a heating rate of 5°C/min. The size and morphology of Cu nanocrystals were observed with a FEI QUANPA200 scanning electron microscope (SEM; QUANPA200, FEI, Hillsboro, OR, USA) and a Japanese Electronics H-700 transmission electron microscope (TEM; Hitachi, Ltd, Chiyoda-ku, Japan) with an operating voltage of 160 kV. X-ray diffraction (XRD) patterns were recorded on a Philips X’pert Pro X-ray diffractometer (PANalytical B.V., Almelo, The Netherlands) equipped with graphite monochromatized Cu Kα radiation (*λ* = 0.15406 nm).

## Results and discussion

Cu nanocrystals were achieved by heating cupric tartrate under high-purity gas in the horizontal furnace. The decomposition process is evident from thermoanalysis, and the size and morphology of Cu nanocrystals can be controlled by adjusting reaction temperature and time.

### Decomposition process

**Figure 1:**
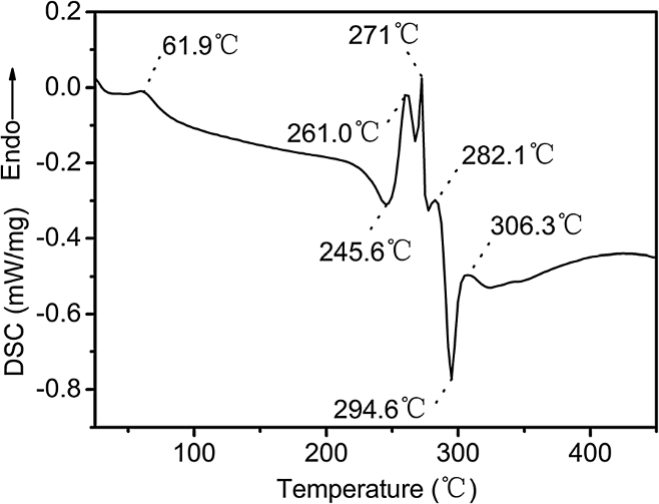
**The DSC curve of the cupric tartrate at heating rate of 5°C/min under N**_**2**_ **.**

Figure 1 shows the differential scanning calorimetry (DSC) curve for cupric tartrate. As seen from the figure, the DSC curve contains four endothermic peaks (261.0°C, 271°C, 282.1°C, and 306.3°C) and two exothermic peaks (245.6°C and 294.6°C). There is also a peak locating at 61.9°C, which is attributed to the dehydration of the crystal water. These results are slightly different from those reported by Qin et al. who reported an endothermic peak at 266.4°C and two exothermic peaks at 290.9°C and 309.3°C [26]. This may be due to the different heating rate used during the DSC experiments. Qin et al. used a heating rate of 10°C/min, which is higher than the one used here. It may cost a longer time to reach the equilibrium of reactions at a higher heating rate.

As illustrated in Figure 1, there are four endothermic peaks on the DSC curve of cupric tartrate. This indicates that the decomposition of cupric tartrate may be a complex process and proceed in several steps, which is in good agreement with the results of Schmid and Felsche [27] who used the same heating rate as our experiments. Schmid and Felsche found that decomposition of cupric tartrate produced numerous of species, including CH_2_, H_2_O, CO, C_2_H_2_, CH_2_O, O_2_, C_2_H_2_O, CO_2_, C_2_H_2_O_2_, C_2_H_4_O_2_, C_2_H_2_O_3_, C_4_H_4_O_4_, and C_4_H_4_O_6_. As for this experiment, the exothermic peaks at 245.6°C and 294.6°C are most likely due to the conversion of C_4_H_4_O_6_^2−^ into an isomeride with a hexatomic ring and the oxidation of Cu species, respectively [27]. The decomposition of tartrate diradical to C_2_H_2_O_3_ could be the origin of the endothermic peak at 261.0°C. Further decomposition of C_2_H_2_O_3_ to formaldehyde and CO_2_ may be responsible for the endothermic peak at 271°C. The endothermic peak at 282.1°C may be due to the decomposition of C_4_H_4_O_6_^2−^ to C_4_H_4_O_4_ and O_2_. The endothermic peak at 306.3°C may be resulted from the decomposition of C_4_H_4_O_4_ to CH_2_, H_2_O, CO, C_2_H_2_, and CH_2_O.

**Figure 2:**
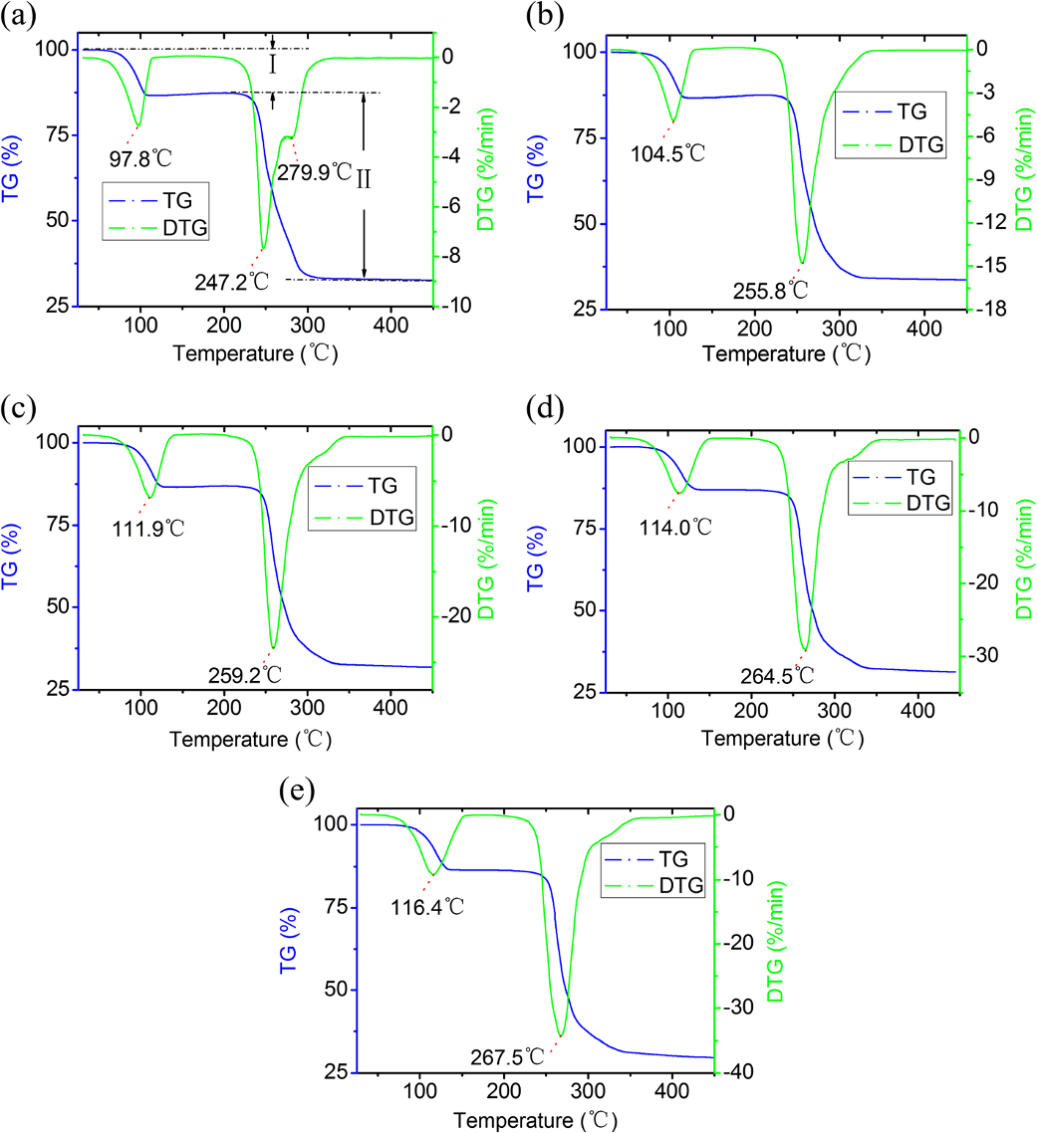
**TG/DTG curves of cupric (II) tartrate at different heating rates. (a)** 5°C, **(b)** 10°C, **(c)** 15°C, **(d)** 20°C, and **(e)** 25°C/min.



**Figure 3:**
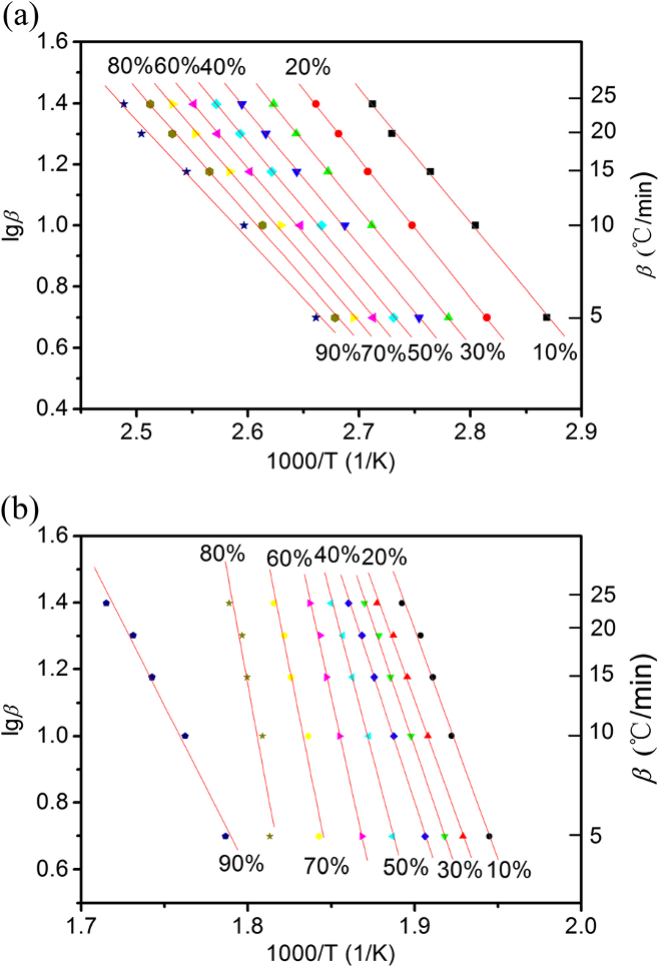
**lg** ***β*** **-1,000 T** ^**−1**^ **relationship of cupric tartrate at decomposition stages: (a) I and (b) II.**

Figure 2 demonstrates the thermogravimetric (TG) and differential thermogravimetric (DTG) curves at different heating rates (5°C, 10°C, 15°C, 20°C, 25°C/min). From these curves, the temperatures for the dehydration of crystal water mainly range from 97.8°C to 116.4°C, and the temperatures for the decomposition process locate between 247.2°C and 267.5°C. The kinetic properties of the decomposition process were analyzed by employing the Flynn-Wall-Ozawa (FWO) method [28]:

where, *β*, *R*, *T*, *A*, *E*, and *G*(*α*) represent the different heating rates, the ideal gas constant, the thermodynamic temperature, the pre-exponential factor, the activation energy, and the mechanism kinetic equation with integral form, respectively. With different heating rates, the linear relationships can be performed by plotting the logarithm lg*β* as a functional of the reciprocal of temperature, 1/T, with the decomposition rate *α* ranging from 0.1 to 0.9. The activation energy can be calculated from the slope of the linear relationships. We divided the overall decomposition process into two stages, including dehydration (stage I) and decomposition (stage II). The matching lines during stage I have good coefficients of association close to 1, with a standard error of 0.0111 on average (Figure 3a and Table 1). This indicates that stage I can be described by one kinetic equation. On the other hand, stage II cannot be described by one kinetic equation, as the slopes obtained from the linear regression change (Figure 3b and Table 2). This may be due to that generations of tartrate diradical and fragments of C_2_H_2_O_2_, C_2_H_4_O_2_, C_2_H_2_O_3_, C_4_H_4_O_4_, and C_4_H_4_O_6_ break the linear relationships.

**Table 1:** **Related dynamic parameters of cupric (II) tartrate at decomposition stage I using FWO method**

Parameters	Values								
Decomposition rate *α*	0.1	0.2	0.3	0.4	0.5	0.6	0.7	0.8	0.9
*E*_*a*_ (kJ/mol)	79.8	82.6	80.7	79.5	79.1	78.1	77.0	75.1	70.9
Coefficients of association *r*	0.9990	0.9998	0.9999	0.9998	0.9996	0.9996	0.9993	0.9987	0.9966
Standard deviation	0.0140	0.0046	0.0037	0.0058	0.0089	0.0095	0.0117	0.0160	0.0263

**Table 2:** **Related dynamic parameters of cupric (II) tartrate at decomposition stage II using FWO method**

Parameters	Values								
Decomposition rate *α*	0.1	0.2	0.3	0.4	0.5	0.6	0.7	0.8	0.9
*E*_*a*_ (kJ/mol)	250.6	251.9	268.8	280.2	347.1	414.4	453.8	495.6	179.5
Coefficients of association *r*	0.9970	0.9985	0.9991	0.9992	0.9980	0.9979	0.9804	0.9610	0.9934
Standard deviation	0.0247	0.0176	0.0133	0.0125	0.0203	0.0209	0.0628	0.0882	0.0367

Further analysis of the most probable kinetic equation is performed by the Malek method [29]. The standard curve (1) and the experimental curve (2) are shown as follow:
12

**Figure 4:**
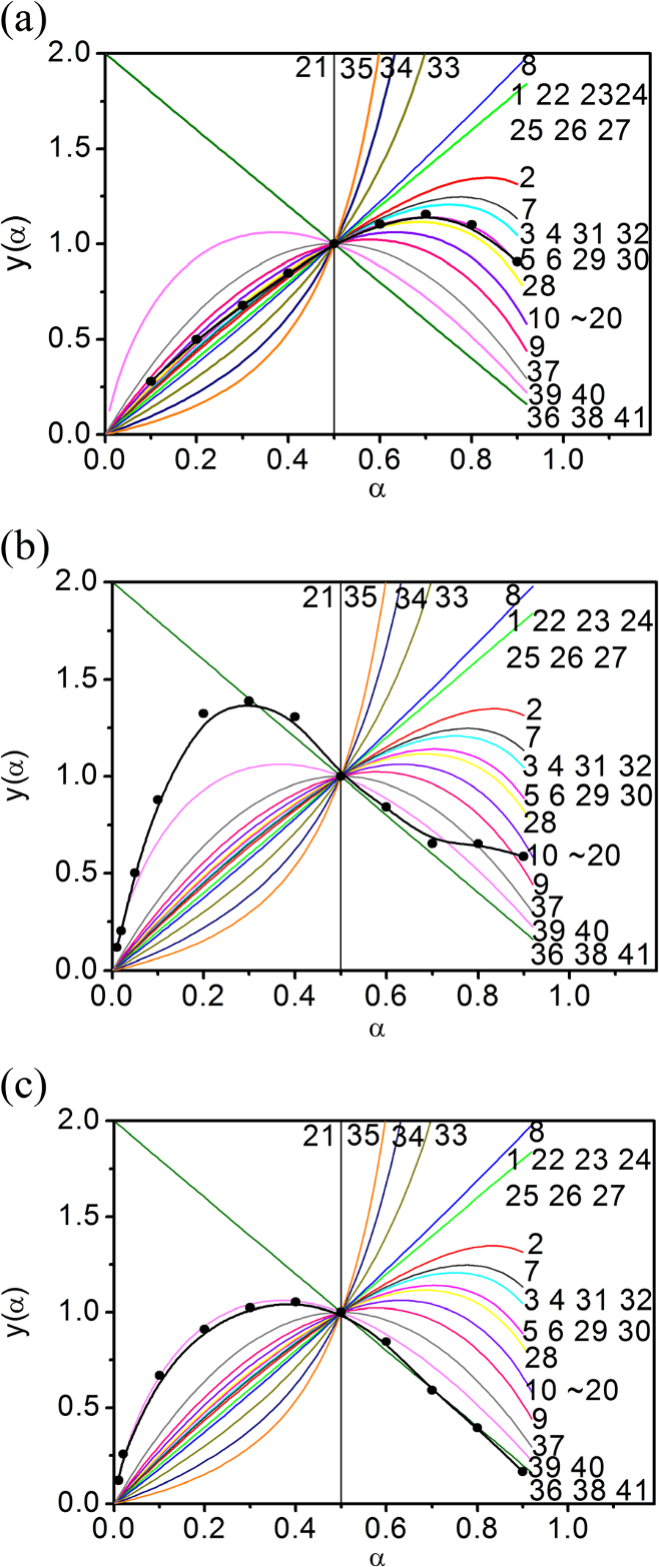
**Experimental result and** ***y*** **(** ***α*** **) −** ***α*** **standard curves of cupric (II) tartrate.** Decomposition stage I **(a)** 5°C ~ 25°C/min and stage II **(b)** 5°C/min and **(c)** 10°C ~ 25°C/min.

As shown in Figure 4a, there is a good fitness between the standard curve and the experimental curve for the dehydration stage. This indicates that the kinetic equation of the dehydration stage is *G*(*α*) = 1 − (1 − *α*)^1/3^ with integral form and *f*(*α*) = 3(1 − α)^2/3^ with differential form, respectively. On the other hand, the experimental curve for the decomposition stage keeps to the accelerated curve and does not fit with the standard curve completely under heating rate of 5°C/min (Figure 4b). The kinetic equation for the decomposition stage may be *G*(*α*) = ln*α*^2^. Under heating rates of 10°C to 25°C/min (Figure 4c), the experimental curves of the decomposition stage are the decelerated curves and fit well with the standard curve, and the kinetic equation of the decomposition stage should be *G*(*α*) = (1 − *α*)^−1^.

The activation energies of the dehydration stage calculated from the FWO, Kissinger [30], and Starink [31] methods are 94.24, 93.07, and 92.79 kJ/mol, respectively, while those for the decomposition stage are 177.33, 177.88, and 177.65 kJ/mol, respectively. The as-calculated results have the approximate value using the three kinds of methods. Thus, the above thermogravimetry analysis by FWO method is reasonable. The kinetic parameters for the decomposition process of cupric tartrate are summarized in Table 3. Based on the above analyses, we would suggest the following possible mechanism:

**Table 3:** **Related dynamic parameters of cupric (II) tartrate using FWO method**

Parameters	Values		
	***E***_***a***_ (kJ/mol)	lg ***A***	***G*** ( ***α*** )
Decomposition stage I	94.24	15.35	1 − (1 − *α*)^1/3^
Decomposition stage II	177.33	20.68, (*α* < 0.5)	ln*α* ^2^, (*α* < 0.5)
		20.63, (*α* > 0.5)	(1 − *α*)^−1^, (*α* > 0.5)

34

These results are in good agreement with the results of the decomposition of cupric tartrate and the formation of tartrate fragmentations reported by Schmid and Qin [26, 27].

### Effect of decomposition temperatures

**Figure 5:**
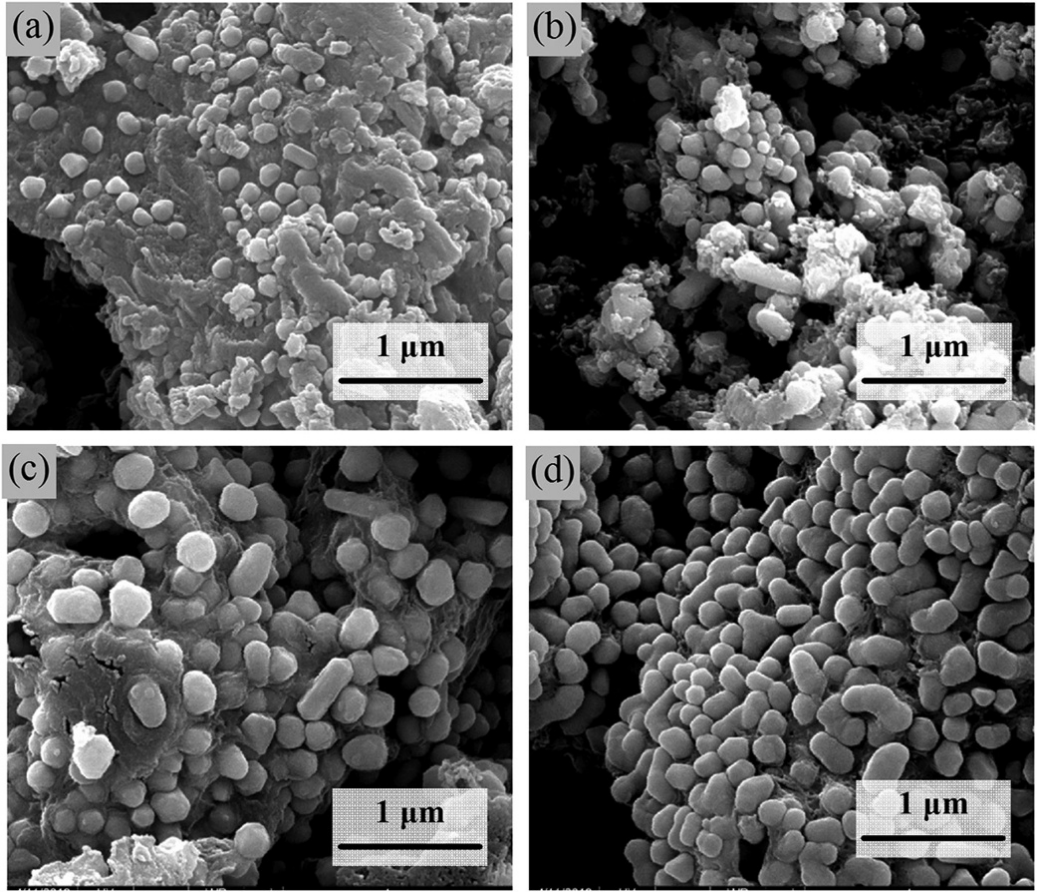
**SEM images of product after the decomposition of the cupric tartrate at different temperatures. (a)** 200°C, **(b)** 250°C, **(c)** 350°C, and **(d)** 400°C.

As reflected by the SEM images shown in Figure 5, the Cu nanocrystals obtained from the decomposition of copper tartrate exhibit rather distinguished shapes in the range from 200°C to 400°C. As described in Figure 5a, microparticles are the main decomposition products at 200°C. As the temperature increases to 250°C, the size of the Cu nanocrystals increases remarkably and some floccules can be found among Cu nanocrystals (Figure 5b). Decomposition at 350°C results in a decrease of the floccules, implying a higher purity of the Cu nanocrystals. The Cu nanocrystals become larger with the temperature increases to 350°C and 400°C, as shown in Figure 5c, d. These results indicate that lower decomposition temperatures favor producing Cu nanocrystals with smaller sizes but lower purity. Higher temperatures are favorable for forming Cu nanocrystals with higher purities, but aggregations of the nanocrystals can easily occur.

**Figure 6:**
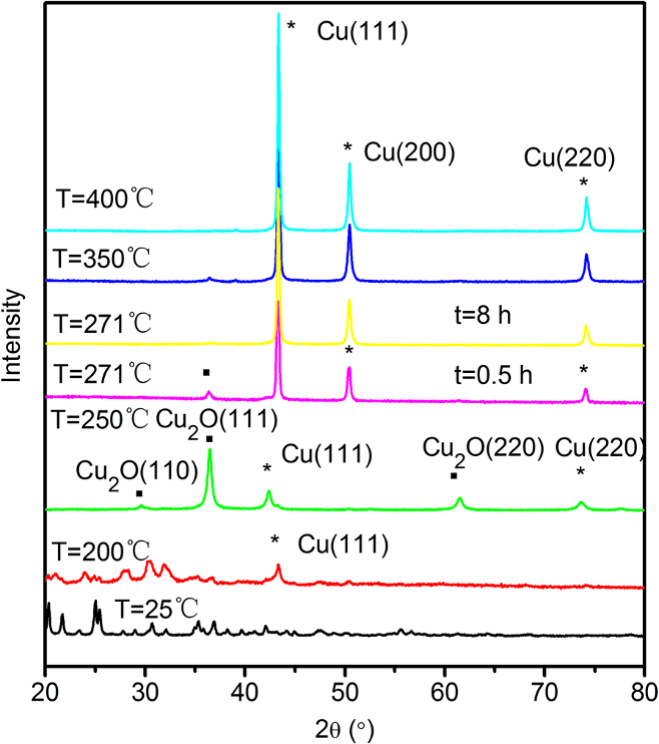
**XRD patterns of products after the decompositions of the cupric (II) tartrate at different temperatures.**

The XRD patterns of the decomposition products at different temperatures are plotted in Figure 6. On the XRD pattern, many characteristic diffraction peaks of cupric tartrate between 13° and 55° are clearly observed, in good agreement with the standard cupric tartrate diffraction pattern (ICDD, PDF file No. 03-065-9743). After heating at 200°C, the diffraction peaks of the floccules and Cu(111) appear on the XRD pattern. As the temperature increases to 250°C, five peaks at 29.6°, 36.5°, 42.4°, 61.4°, and 73.7° are present and are attributed to Cu_2_O(110), Cu_2_O(111), Cu_2_O(220), Cu(111), and Cu(220), respectively. This suggests that the floccules disappear at 250°C. On the XRD pattern of the sample obtained from the decomposition processes at 350°C and 400°C, the peaks assigned to Cu_2_O(110), Cu_2_O(111), and Cu_2_O(220) disappear. It should be noted that there are three peaks 43.3°, 50.5°, and 74.1°, which are due to the Cu metal, on the XRD pattern of the sample formed from decomposition at 400°C. It may caused by the reduction of Cu_2_O by CO and C_2_H_2_ generated from the decomposition of cupric tartrate.

### Effect of reaction time

**Figure 7:**
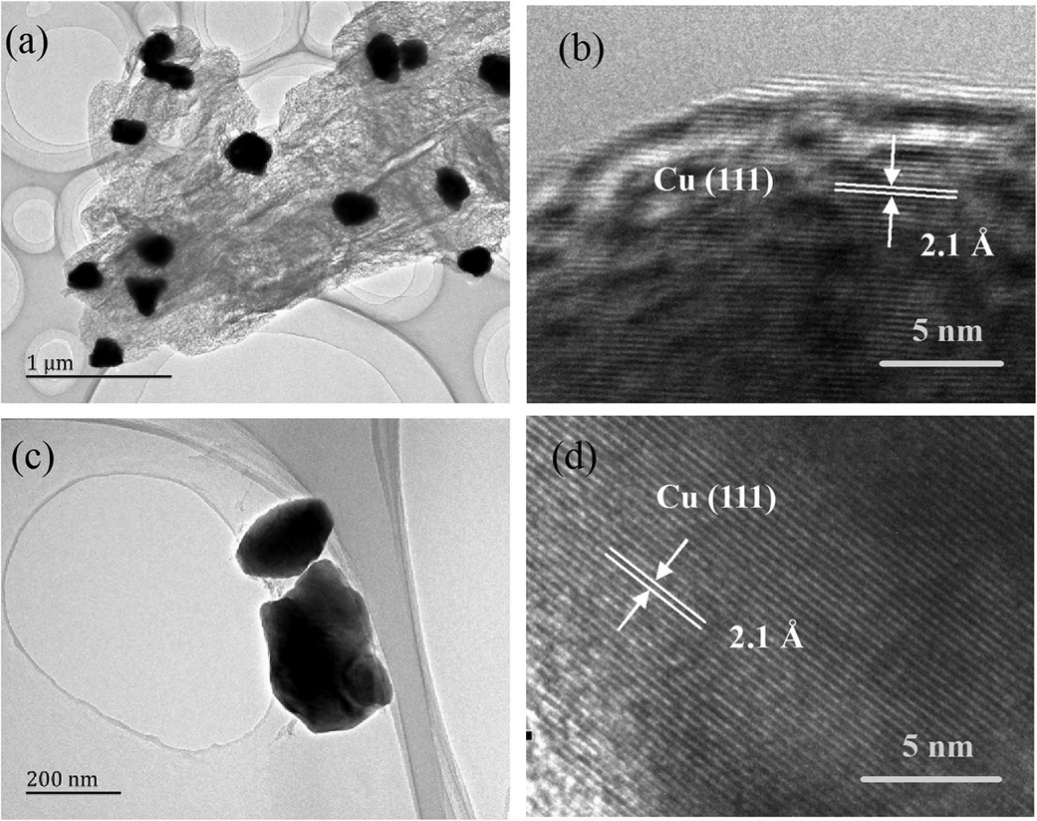
**TEM images of copper particles obtained from the decomposition of cupric tartrate at 271°C under Ar. (a, b)** 0.5 h and **(c, d)** 8 h.

In this section, we will discuss the effect of the reaction time on the properties of the Cu nanocrystals by fixing the decomposition temperature at 271°C. As shown in Figure 6, the sample produced from the decomposition at 271°C has a higher purity. At the same time, the decomposition temperature, i.e., 271°C, is lower than the temperatures which can induce serious aggregations. From the XRD patterns shown in Figure 6, the sample obtained from the decomposition at 271°C for 8 h is much higher than that obtained decomposition at 271°C for 0.5 h. With quantitative analysis of the sample after decomposition for 0.5 h by HighScore® software, the moral ratio of pure Cu nanocrystals is 93%, while that of Cu_2_O is 7%. When the decomposition time was elongated to 8 h, Cu_2_O disappears, as reflected by the XRD patterns plotted in Figure 6. Figure 7a, b, c, d shows the TEM images of the samples from the decomposition at 271°C for 0.5 and 8 h. Lattice fringe with *d* = 2.1 Å confirms the formation of pure Cu nanocrystals. The floccule stuff is also observed in the products as shown in Figure 7a, which is in agreement with the results of SEM characterization.

### First-principles study of the decomposition of cupric tartrate

**Figure 8:**
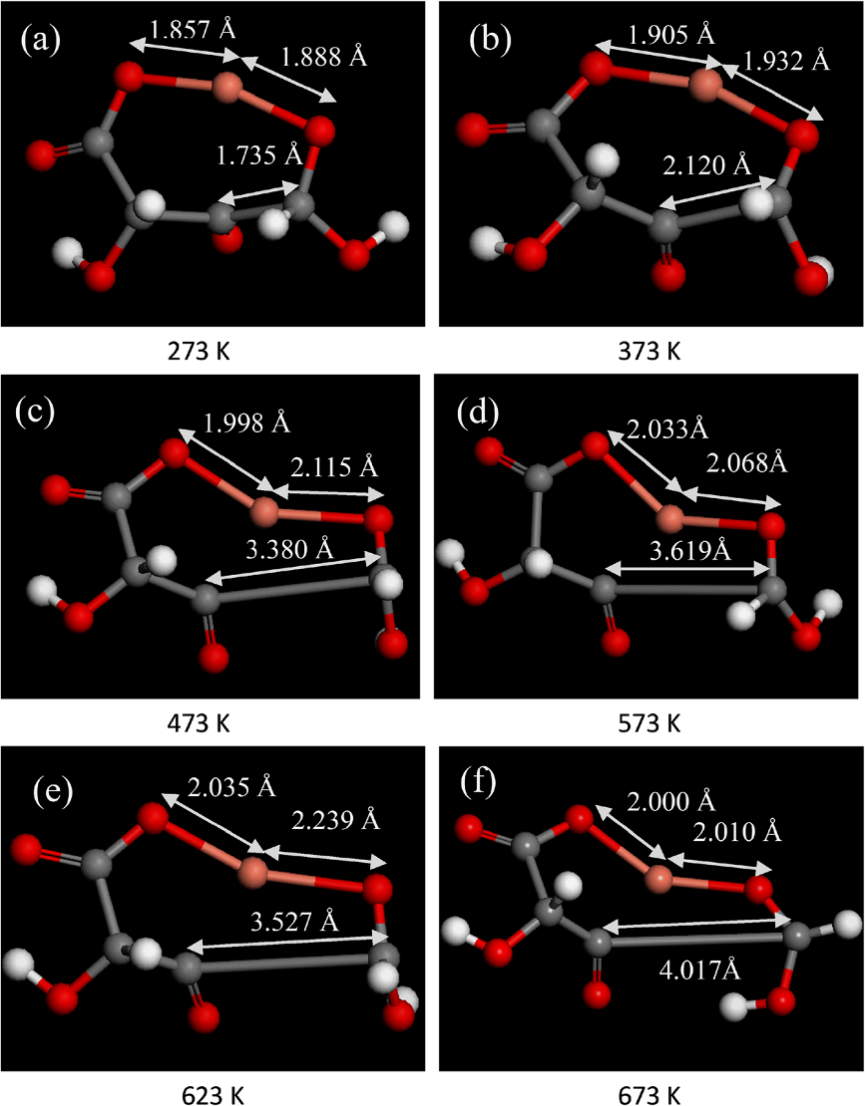
**The images of optimization conformation of cupric tartrate (CuC**_**4**_ **H**_**4**_ **O**_**6**_ **).** After molecular dynamic course at the initial temperature from 273 ~ 673 K. **(a)** 273 K, **(b)** 373 K, **(c)** 473 K, **(d)** 573 K, **(e)** 623 K, and **(f)** 673 K.

In order to reveal the impetus of copper formation, it is necessary to investigate reaction mechanism of cupric tartrate, in particular to the decomposition kinetics, which is directly related to the formation of copper nanocrystals. However, the decomposition process of metallorganics can hardly be measured. We studied the molecular dynamic of cupric tartrate (CuC_4_H_4_O_6_) by density functional theory (DFT) calculations [32] with the generalized gradient approximation (GGA) of PBE [33], as implemented in the Dmol^3^ package [34]. The basis sets used in this work were double-numerical quality basis sets with polarization functions (DNP), which is comparable to the Gaussian 6–31 G** basis set in size and quality [35]. The global orbital cutoff scheme was used, and a 4.4 Å was assigned as global orbital cutoff. We optimized all possible isomers and calculated the binding energies. For nonperiodic systems of copper tartrate, only the NVE and NVT ensembles are available. In order to reasonably study the decomposition kinetics, NVT ensembles were used to control the reaction temperature, which fits well with the related experiments. To the energy-favored stable structure of cupric tartrate, molecular dynamic (MD) simulations were performed using the NVT ensemble (i.e., constant number of atoms, constant volume, and constant energy) which allows both the temperature and stress of the system to change during the decomposition. The molecular dynamic simulation was performed at the initial temperature range of 273 ~ 673 K using NVT ensemble, with a time step of 0.1 fs and simulation time of 0.2 ps. The two Cu-O bonds both increase with the temperature increasing from 273 ~ 673 K as shown in Figure 8. Compared with the original Cu-O bond length of 1.868 and 1.848 Å, the elongated bond length has the maximum value of 2.035 and 2.239 Å at 623 K, respectively, which demonstrates the formation of Cu atom. Besides, the length of a C-C bond elongates from 1.727 to 4.107 Å as the temperature increases up to 673 K. And then, molecular of cupric tartrate decomposes into a Cu atom, HCOOH, and C_3_O_4_H_2_ fragment. It can be speculated that Cu atoms generate from the cupric tartrate prior to the full decomposition of fragments which is in agreement with the fact that Cu nanocrystal and fragments coexist at relatively low temperatures.

## Conclusions

In the present work, high-purity Cu nanocrystals were successfully synthesized from the decomposition of cupric tartrate at 271°C for 8 h under the atmosphere of Ar. The size and purity of the Cu nanocrystals depend on the decomposition temperature and time. Higher temperatures are favorable for the production of highly pure Cu nanocrystals but induce serious aggregations. Longer decomposition time leads to high-purity Cu nanocrystals of larger size. Kinetic analysis results indicated that the conversion of cupric tartrate to Cu nanocrystals is a two-step process, including dehydration and decomposition steps. It is expected that the efficient but simple decomposition method proposed in the present work will help to open a new way to prepare highly pure Cu nanocrystals with desired sizes and morphologies.
